# Combined climate stressors constrain various mechanisms for thermal tolerance in the scallop *Pecten maximus*

**DOI:** 10.1242/jeb.250291

**Published:** 2025-12-15

**Authors:** Sandra Götze, Charlotte Eymann, Carl J. Reddin, Gisela Lannig, Christian Bock, Hans-Otto Pörtner

**Affiliations:** ^1^ Integrative Ecophysiology, Alfred Wegener Institute Helmholtz Centre for Polar and Marine Research, 27570 Bremerhaven, Germany; ^2^ MARUM – Center for Marine Environmental Sciences, University of Bremen, 28359 Bremen, Germany

**Keywords:** Filtration rate, Heat shock response, OCLTT, Oxidative stress, Climate change, NMR spectroscopy

## Abstract

Unfavourable climatic conditions challenge an animal's performance and fitness. We investigated how cellular homeostasis relates to whole-animal physiology in the marine bivalve *Pecten maximus* under warming (W), warming plus hypercapnia (WHc) or hypoxia (WHo), and the combination of all three drivers (deadly trio, DT). Starting at 14°C, temperatures were increased stepwise by 2°C per 48 h while gill tissue was sampled from experimental exposures to test for indicators of intracellular stress, including lipid peroxidation, protein damage and degradation, apoptosis and heat shock responses. Whole-animal water filtration rate, routine metabolic rate (RMR), haemolymph *P*_O_2__, pseudofaeces ejection, mantle tissue intracellular acidosis and gill tissue antioxidative capacity were measured in W and DT exposures. Filtration peaked at a lower temperature under DT, when high pseudofaeces ejection suggested that ventilation was prioritized over feeding. Warming alone doubled RMR by 22°C, whereas DT increased RMR even further, reaching higher maxima by 20°C. Haemolymph *P*_O_2__ was consistently lower under DT, implying that supply was less able to meet increasing demand. Warming to 26°C stimulated a gill tissue heat-shock response, accumulated ubiquitin conjugates and apoptosis, whereas adding hypoxia or hypercapnia suppressed apoptosis. DT suppressed both heat shock and apoptotic responses, with ubiquitin conjugates and branched-chain amino acids accumulating, and gills showing visible damage. Our findings indicate that climate drivers cumulatively block protection mechanisms, increase protein damage and block protein synthesis, thereby substantially reducing passive thermal tolerance and survival under extremes. The thermal tolerance of scallops is critically reduced under DT conditions, when mechanisms defending passive tolerance are exhausted at lower temperatures.

## Teaser

**Summary:** Multiple climate drivers cumulatively constrain the mechanisms by which the scallop *Pecten maximus* can tolerate warming beyond its thermal optimum, from whole-organism to intracellular responses, with survival implications.

## INTRODUCTION

Extensive combustion of fossil fuels by humans, causing global warming, seawater acidification (IPCC, 2021), and the formation and expansion of oceanic hypoxic zones (Breitburg et al., 2018; IPCC, 2019), may stress marine life through physiological mechanisms. Mechanism-based models can project climate impacts on marine animal fauna (Penn et al., 2018; Penn and Deutsch, 2022). Models that link physiological performance and the energy budget of animal ectotherms to a limited temperature range may be especially promising, such as the concept of oxygen- and capacity-limited thermal tolerance (OCLTT; Pörtner, 2002, 2010; Pörtner et al., 2017), which had some debate during its emergence (see Verberk et al., 2016; Jutfelt et al., 2018). According to the predictions of OCLTT, a progressive mismatch between oxygen supply and demand sets in outside the thermal optimum and beyond thermal limits, called pejus temperatures, causing the animal's energy budget and aerobic performance to fall once outside the thermal optimum. Foraging and feeding capacities are constrained, cellular homeostasis is gradually impaired, and finally passive sustenance, survival and population persistence are limited (see Pörtner and Knust, 2007; Sokolova et al., 2012; Eymann et al., 2020; Pörtner, 2021). The interaction of temperature with additional abiotic drivers such as hypoxia and hypercapnia frequently constrains aerobic performance, narrowing the thermal tolerance range, including the active range between upper and lower pejus temperatures (Walther et al., 2002, 2009; Pörtner et al., 2005; Metzger et al., 2007; Sokolova et al., 2012; Melzner et al., 2013). Under more extreme temperatures, organisms then mobilize cellular protection and repair mechanisms, which enable survival by limiting damage, either through anaerobic metabolic pathways or metabolic depression. However, once above pejus temperatures, the capacity of such mechanisms and, in consequence, tolerance to climate extremes is strictly time limited and passive, because active activities such as roaming and feeding are increasingly constrained (Pörtner, 2010). Bivalves use anaerobic pathways when their shells are temporarily closed, and fully recover thereafter when temperatures are not outside of their thermal range. Towards thermal extremes, meanwhile, anaerobic metabolism covers the net energy deficit emerging from insufficient capacity of aerobic metabolism. Therefore, according to lifestyle and habitat characteristics, the capacity for time-limited passive tolerance to heat stress may vary between taxonomic clades, species and populations, shaping their vulnerability to different climate drivers.

In suspension-feeding bivalves, water pumping through the mantle cavity and over the gills serves both gas exchange and food capture simultaneously. Prioritization of respiration over ingestion and digestion may be a response to climatic stress, which may lead to uneaten food being deposited as pseudofaeces, such as produced when exposed to high seston concentration (Foster-Smith, 1975; Riisgård, 2001; Kooijman, 2006). CO_2_ release via gaseous exchange may support intracellular pH regulation, whereby pH change follows a temperature-dependent curve in ectotherms to maintain the protonation equilibria of proteins and thereby their functioning (Reeves, 1985; Wang and Jackson, 2016).

In response to temperature extremes, the cellular expression of heat shock proteins, antioxidative defence and other responses provide protection and extend survival beyond pejus temperatures (Pörtner, 2010), albeit temporarily, awaiting the return of benign conditions. Antioxidative defences balance the release of reactive oxygen species, which increase with heat stress and can damage macromolecules including proteins and lipids, disrupt cellular functioning, and cause apoptosis (programmed cell death) if damaged proteins are not appropriately removed (Abele et al., 2001; Tomanek et al., 2011; Lesser, 2006; Ali et al., 2020). Hypoxia also activates the ‘heat shock’ response [David et al., 2005 (oyster); Anestis et al., 2010 (mussel); Brokordt et al., 2015 (scallop)], with heat shock protein 70-kDa (HSP70) being a sensitive indicator of thermal and hypoxic stress (Chapple et al., 1998; Fabbri et al., 2008; Anestis et al., 2010). The effects of environmental hypercapnia on the heat shock response vary with the duration of exposure and tissue type (Thompson et al., 2015; Goncalves et al., 2017; Sokołowski and Brulińska, 2018). Pacific oysters exposed to various levels of CO_2_ (1000 versus 2000 µatm *P*_CO_2__) exhibited highly upregulated branchial expression of HSP70 after 7 days of hypercapnia, but significant downregulation resulted after 14 and 28 days (Wang et al., 2016). These cellular responses are hypothesized to initiate acclimatization, thereby shifting thermal tolerance limits with continued or repeated exposure (Pörtner et al., 2017). In marine bivalves, the thermal threshold of the heat shock response therefore shifts with season, habitat (e.g. height on the seashore, in intertidal species) and reproductive status (e.g. Chapple et al., 1998; Fabbri et al., 2008; Brun et al., 2008; Brokordt et al., 2015). An insufficient heat shock response causes a cellular accumulation of damaged, ubiquinated proteins, which may trigger early apoptosis as part of the repair chain, aiming to limit more widespread or escalating tissue damage. Apoptosis mechanisms are highly conserved among phyla (Elmore, 2007; Romero et al., 2015; D'Arcy, 2019), particularly caspases, an enzyme family with a key role in apoptosis (Brentnall et al., 2013). Temperature, hypercapnia or pollutants can also induce pro-apoptosis in marine bivalves (Kefaloyianni et al., 2005; Wang et al., 2016; Rahman and Rahman, 2021; Vogeler et al., 2021).

Projected future changes in the climate and ocean geochemistry resemble those coinciding with ancient mass extinctions such as the Permian–Triassic mass extinction (Sun et al., 2012; Song et al., 2013), which was triggered by massive release of greenhouse gases from Siberian Trap volcanism. Evolutionary history may conserve physiological stress mechanisms alongside morphological features (i.e. uniformitarianism), such that palaeobiological findings from ancient rapid global warming crises may help understand the modern crisis, and vice versa (Pörtner et al., 2004; Knoll et al., 2007; Calosi et al., 2019; Reddin et al., 2020; Kiessling et al., 2024). Using this principle, the end-Permian distribution of extinction victims has been linked to the global distribution of marine organisms vulnerable to multiple climatic change drivers, although the tolerance capacity of invertebrates was not well represented (Penn et al., 2018). The class Bivalvia, one of the largest groups of modern coastal benthic invertebrates with high modern ecological and economic value (Smith et al., 2014), suffered widespread extinctions and a collapse in taxonomic richness during the Permian–Triassic (Tu et al., 2016; Nakazawa and Runnegar, 1973). This included scallops (Pectinidae), many species of which cannot fully close their shells, have eyes, and have varying abilities to swim short distances by valve clapping and jet propulsion (Wilkens, 2006; Tremblay and Guderley, 2014). These traits are strong in *Pecten maximus*, where they are fuelled by a high energy turnover, supported by higher levels of mantle cavity *P*_O_2__ and higher standard metabolic rates than in less active, blind benthic molluscs (Abele et al., 2010; Schalkhausser et al., 2013, 2014). As a result, scallops are hypothesized to be active, aerobic and less tolerant to environmental fluctuations than bivalve clades that commonly inhabit intertidal zones, e.g. ostreoids and mytiloids (see e.g. Eymann et al., 2020; Götze et al., 2020).

Here, we explored the linkage of the cellular stress response and its activation with whole-organism physiology and performance in *P. maximus* under cumulative warming alone and combined with hypoxia and/or hypercapnia. We hypothesized that additional hypoxia and/or hypercapnia would narrow the thermal tolerance range, revealing a higher vulnerability in *P. maximus* than in the sympatric but intertidal *Ostrea edulis* (parallel works: Eymann et al., 2020; Götze et al., 2025; our unpublished results). We report filtration and respiration rates, haemolymph (‘extracellular’) oxygen partial pressure measurements (*P*_O_2__, as *Pecten* does not have a blood pigment) and pseudofaeces production as potential signs of organism-level disturbance. We report these alongside cellular indicators of stress within gill or mantle tissue, including lipid peroxidation, changes in intracellular pH, protein damage and degradation, heat shock response, antioxidative capacity and apoptosis.

## MATERIALS AND METHODS

### Experimental design and animals

All experiments were carried out using *Pecten maximus* (Linnaeus 1758) as described in Götze et al. (2020). In brief, adult specimens were caught in the estuary of Vigo, Spain (∼42°14′46.6*″N*, 8°44′18.5″W), by SCUBA divers of the Biological Station of Toralla (ECIMAT, University of Vigo) in autumn 2017 (at ∼18°C and ∼33 PSU) and spring 2018 (at ∼16°C and ∼33 PSU). Upon arrival at the Alfred Wegener Institute (Bremerhaven, Germany), scallops were introduced to the institutional aquarium systems and acclimated gradually to 14°C and ∼34 PSU salinity over at least 4 weeks. Every second day, scallops were fed (minimum of 3000 cells ml^−1^ SW) with a mixture of commercial algal blend (Nyos, PhytoMaxx) and self-cultivated microalgae including *Rhodomonas* sp., *Phaeodactylum tricornutum*, *Chaetocerus* sp. and *Isochrysis galbana*. Adult scallops had an average length and width of 10.4±1.1 and 10.8±1.3 cm, respectively, and weighed 163.4±46.0 g. *Pecten maximus* were carefully cleaned of epibionts and distributed among four experimental setups: warming alone (normoxia/normocapnia control; W), warming plus hypoxia (WHo), warming plus hypercapnia (WHc) and the combined exposure of all three stressors (deadly trio, DT). Seawater was kept fully oxygenated (>90%, or *P*_O_2__ between 19.2 and 20.6 kPa, dependent on temperature) in both normoxic exposures and was reduced to 55–60% (*P*_O_2__ between 10.7 and 13.5 kPa, dependent on temperature) in both hypoxic exposures. We consider such levels as mildly hypoxic and above critical *P*_O_2__ when within the thermal window of the species (Brand and Roberts, 1973) (Table S1). In the normocapnic exposures, CO_2_ levels were kept as low as possible (∼550 µatm) whereas levels were increased to 1800 µatm CO_2_ in the WHc and DT exposures, values that can be reached and surpassed in intertidal environments.

We used a cumulative heat ramp, where exposures started at 14°C and temperature was increased stepwise by 2°C per 48 h, allowing physiological functions to stabilise at each step, until all scallops had died. For the cellular responses, tissue samples were taken at 14°C, 18°C, 22°C and 26°C and shock frozen in liquid nitrogen (target *N* per temperature=8, though limited by survival). In the W, WHc and DT exposures, scallops had died upon reaching 28°C, whereas under WHo, 13 out of 17 residual scallops died during transition from 24°C to 26°C. Full mortality details are reported in Götze et al. (2020). Cumulative heat ramp experiments conflate the effects of temperature per se with the cumulative duration the organisms are held under exposure conditions. Time has a different meaning for tolerance at different temperatures of the thermal performance curve, from virtually unlimited duration in the thermal optimum to survival becoming progressively more time limited at high or low extreme temperatures. The experimental protocol thereby elicits and distinguishes where passive replaces active thermal tolerance. Cumulative warming is also more realistic than warming by larger, sudden steps, which avoids conflation with an organism's overshoot or undershoot response to sudden change. The time–temperature conflation, especially in animals that were additionally exposed to hypoxia and/or hypercapnia, has important implications for understanding the observed responses, because duration above upper (or below lower) pejus temperatures gradually exhausts an organism's tolerance.

### Whole-organism responses

For logistical reasons, whole-organism responses were recorded from scallops exposed to W and DT treatments only, collected in 2018. Randomly selected scallops were identified by labelling or by separating them from the others to ensure repeated measurements of filtration rate and, separately, routine metabolic rates (RMRs) on the same individuals (see below). Mortality in allocated scallops prevented filtration rate measurements in DT exposures at 26°C and 28°C and RMR measurements in both treatments at 26°C and 28°C.

#### Filtration rate

Filtration rates were determined as described in Eymann et al. (2020), by calculating the decrease of *Rhodomonas* sp. density over time standardized to soft body dry mass. At each temperature step, the same individually labelled scallops were placed in temperature-controlled cylindrical 2.5 litre tanks aerated with the treatment-specific gas mixtures. After 1 h of recovery, *Rhodomonas* sp. was added (final density 30,000 cells ml^−1^ to ensure sufficient algae provision for maximal filtration rate) and samples were taken after 0, 15 and 30 min. Gentle bubbling prevented sedimentation of algal cells, which was found to be negligible in a control that used separate 2.5 litre tanks fitted with empty *P. maximus* shells. Algae density was measured with a Multisizer 3TM Coulter Counter (Beckman, USA) fitted with a 100 µm aperture tube. Dry mass of soft body mass was recorded after drying for 48 h at 80°C when constant mass was reached.

The filtration rate (*F*) was calculated according to Coughlan (1969) and expressed as rate per gram dry mass (l h^−1^ g^−1^ DM):
(1)

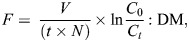
where *V* is the water volume of the experimental tank (l), *t* is time (h), *n* is the number of individuals per tank (here, *N*=1), *C*_0_ and *C_t_* are the algal densities (cells ml^−1^) at time 0 and time *t*, respectively, and DM is the soft body dry mass (g). Additionally, pseudofaeces production was informally observed and recorded at the end of each measurement (after 30 min) as ‘none’, ‘some’ or ‘only’ pseudofaeces, which was ordered as a rough ratio of pseudofaeces to faeces over these individuals (0% suggesting digestion is prioritized, 50% suggesting stress, 100% suggesting digestion is being skipped)*.* Together, these variables may indicate when ventilation is prioritized over feeding and digestion.

#### Routine metabolic rate

Online measurements of RMR were performed using flow-through respirometry. Respiration chambers (400 ml) were placed into temperature-controlled experimental tanks that were aerated with the treatment-specific gas mixture (see section ‘Experimental design and animals’). Temperature was increased once every 48 h by 2°C and kept within ±0.5°C. Practically, this temperature increase was not immediate but stabilized within a 5 h period overnight. The summary of the water parameters can be found in Table S1. Every second day during treatment temperature increase, the chambers were cleaned and animals were fed the self-cultivated algal mixture (as above, but here 10,000 cells ml^−1^). After ∼12 h at the experimental target temperature, chamber lids were closed, connected to the flow-through system, and oxygen consumption was monitored (for the remaining ∼36 h) using O_2_ optodes (Microx TX3 and Fibox3, PreSens, Neuweiler, Germany), calibrated at the respective temperature. Flow rates were adjusted to ensure continuous air saturation levels of ≥75% (normoxia) during the W treatment and ≥45% (hypoxia) during the DT treatment, and varied between 20.3 and 85.0 ml min^−1^ depending on temperature. Bacterial respiration was measured in empty chambers and was found to be negligible. At the end of the experiment, scallops were dissected and soft body dry mass was determined. RMR, measured as oxygen consumption rate *Ṁ*_O_2__, was evaluated following Lannig et al. (2008) and expressed as rate per gram dry mass (µmol O_2_ h^−1^ g^−1^ DM):
(2)

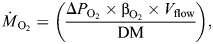
where Δ*P*_O_2__ is the difference in oxygen partial pressure between inflowing and outflowing water (torr), β_O_2__ is the oxygen solubility coefficient in seawater at the respective temperature (µmol O_2_ l^−1^ torr^−1^, taken from Boutilier et al., 1984), *V*_flow_ is the flow rate (l h^−1^) and DM is the soft body dry mass (g).

The treatment-specific effect on temperature-induced changes in RMR was assessed through the *Q*_10_ temperature coefficient calculated as:
(3)

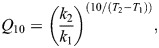
where *k*_1_ and *k*_2_ are the mean RMR measured at temperature *T*_1_ and *T*_2_, respectively.

#### Haemolymph oxygen partial pressure

After ∼38 h at the experimental target temperature of each temperature step, randomly selected scallops were dissected on ice for the measurement of haemolymph oxygen partial pressure (*P*_O_2__) and for tissue sampling (see Götze et al., 2020). Haemolymph samples were taken from the cardiocoelom with gas-tight syringes and measured immediately at the respective temperature using a blood gas analyser (modified MT 33, Eschweiler & Co, Kiel, Germany) connected to a cooling system. The instrument was calibrated at the respective temperature prior to sampling.

### Cellular responses

Cellular responses were recorded for all treatments, except for intracellular pH (pH_i_) and oxygen radical absorbance capacity which, like whole-organism responses, were recorded from W and DT treatments only.

#### Intracellular pH

Disturbances of pH_i_ were assessed in scallop mantle tissue using the homogenate technique developed by Pörtner et al. (1990). In brief, mantle tissue was ground with mortar and pestle under liquid nitrogen. Approximately 100 mg of tissue was added to 0.2 ml of medium (0.16 mol l^−1^ potassium fluoride, 0.002 mol l^−1^ nitriloacetic acid, pH 6.8, 0°C) in a 0.5 ml tube. The tube was stirred quickly with a needle to remove air bubbles, filled up with medium and closed. The homogenate was sonicated twice for 45 s (at −7°C) and immediately centrifuged at 15,500 ***g*** for 50 s (at 4°C). pH_i_ was measured with a microelectrode (Mettler-Toledo) calibrated with high-precision pH buffers (Mettler-Toledo). The electrode was connected to a Power Lab (ADInstruments) via the pH meter, and the pH was recorded using LabChart. The measurements took place in a temperature-controlled system at the respective sample temperature, with the following settings: voltage=500 mmol l^−1^, resolution=150 mV and speed=10/s. The pH was considered as stable after a constant voltage for 1 min.

#### Oxygen radical absorbance capacity

The oxygen radical absorbance capacity (ORAC) of scallop gills was detected using a modified protocol according to Ou et al. (2001) and Furuhagen et al. (2014). Ground tissue was homogenized in 10 mmol l^−1^ potassium phosphate buffer (pH 7.4), sonicated on ice and centrifuged thereafter for 15 min at 13,000 ***g*** (4°C). The assay was performed in microplates using 0.106 nmol l^−1^ fluorescein as a probe and 152.6 mmol l^−1^ 2.2-azobis(2-amidinopropane)-dihydrochloride (AAPH) as a producer of the radicals. On each plate, 0–100 µmol l^−1^ Trolox was used as a standard. Diluted supernatants were directly applied to the prepared microplate containing 150 µl fluorescein. The reaction was started by adding 30 µmol l^−1^ AAPH and recorded in a Fluoroskan Ascent FL microplate reader (Thermo Fisher Scientific; excitation: 485 nm/emission: 538 nm) at 25°C until the reaction was completed (∼3 h). Thereafter, the area under the curve (AUC) was calculated for each sample using SigmaPlot (12.0, Systat Software) and converted to the µmol l^−1^ Trolox equivalent. All values were corrected for the respective soluble protein content per sample and data are expressed in nmol Trolox equivalent per µg protein. Protein content was determined after Bradford (1976) using bovine serum albumin as standard.

#### Lipid peroxidation

Malondialdehyde (MDA) was measured as a marker for lipid peroxidation in scallop gills using a thiobarbituric acid test developed by Uchiyama and Mihara (1978), modified after Abele et al. (2002). Briefly, ∼75 mg tissue was homogenized with Precellys 24 (Bertin Technologies, France; twice for 15 s at 5000 rpm and 4°C) in 0.2% phosphoric acid (1:5 wet tissue to acid volume ratio). After homogenization, phosphoric acid (2%) was added to a final concentration of 1.1%. Each sample was divided into two subsamples (200 µl each): one was supplemented with 1% thiobarbituric acid (in 50 mmol l^−1^ NaOH) and the other with an equal volume of 3 mmol l^−1^ HCl. Using pH paper, all samples were tested to have a pH around 1.6 before they were incubated for 60 min at 100°C using a water bath. Thereafter, 1 ml pure butanol was added, vortexed (40 s) and centrifuged for 5 min at 1000 ***g*** at room temperature. The upper phase was collected and centrifuged again for another 5 min at 14,000 ***g***. The supernatant was measured in triplicate at 530 nm in a microplate reader (Berthold). The absorbance was quantified by a standard series using MDA-bis-acetate. Levels of MDA were expressed in nmol g^−1^ wet mass gill tissue.

#### Protein damage and protection

Soluble proteins were extracted from scallop gills by homogenizing the tissue in 5-fold volume (vol/w) of ice-cold homogenization buffer [10 mmol l^−1^ Tris-HCL, 10 mmol l^−1^ KCL, 1.5 mmol l^−1^ MgCl_2_, 0.5 mmol l^−1^ PMSF, 1 mmol l^−1^ Na_3_VO_4_ and 5 µg ml^−1^ full protease inhibitor mix (Sigma, P8340)]. Homogenization was carried out by Precellys 24 for 20 s at 6000 rpm and 4°C (Bertin Technologies, France). Homogenates were centrifuged (15 min, 13,000 ***g***, 4°C) and the soluble protein concentration in the supernatant was determined after Bradford (1976; bovine serum albumin as standard). All samples were diluted with SDS-loading buffer (0.125 mol l^−1^ Tris-HCL, pH 6.8, 4% SDS, 20% glycerol, 0.01% Bromophenol Blue, 2.5% ß-mercaptoethanol, after Laemmli, 1970) to a final concentration of 1 µg protein µl^−1^. Samples were heat-denatured for 10 min at 95°C (Laemmli, 1970). For electrophoresis, 10 µg protein per sample was randomly applied to 10% (w/vol) acrylamide gels and run at 200 V for ∼45 min using Bio-Rad Mini-Protean chambers. Each gel contained a protein marker and a reference sample for quantification of signals across gels. After electrophoresis, proteins were transferred on polyvinylidene difluoride membranes using Mini Trans-Blot^®^ Cell systems (Bio-Rad) filled with ice-cold transfer buffer (25 mmol l^−1^ Tris, 192 mmol l^−1^ glycine, 20% pure methanol) according to the manufacturer's guidelines (2 h at 200 mA per system). Unspecific binding sites on membranes were blocked for 1 h at room temperature using 5% (w/vol) fat-free milk in TBST wash buffer [20 mmol l^−1^ Tris-HCL (pH 7.5), 137 mmol l^−1^ NaCl, 0.1% Tween-20 (vol/vol)]. Primary antibodies for HSP70 (1:5000, MA3-006; Thermo Fisher Scientific) and ubiquitin (1:2000, P4D1, Enzo Life Sciences) were used in 5% fat-free milk in TBST overnight at 4°C and prepared freshly before each use. On the next day, membranes were washed thoroughly with TBST and incubated for 2 h at room temperature in anti-mouse secondary antibody coupled to horseradish-peroxidase (1:40,000, NA931V, GE Healthcare, Munich, Germany). Chemiluminescence was detected by enhanced chemiluminescence (ECL) solution (GE Healthcare, Munich, Germany) using a cooled charge-coupled image reader (Fujifilm Intelligent dark box LAS-1000). Calculation of signal intensity (given as arbitrary units=LAU) was performed using the AIDA Image Analyzer v. 3.52 software (Raytest, Straubenhardt, Germany). The HSP70 antibody detects multiple members of its family. We detected two isoforms (∼69–70 kDa range, though one was very faint) and summed intensities of both for quantification. The antibody against ubiquitin detects ubiquitin conjugates of any protein size, resulting in a typical binding smear on the blot. In this case, the intensity of the whole lane was quantified. Intensities were normalized against the reference sample run on all gels.

#### Branched-chain amino acids

Quantities of branchial, single proteinogenic branched-chain amino acids (BCAAs; namely, valine, leucine and isoleucine) were derived and summed from our previous study (Götze et al., 2020). Briefly, metabolic profiles were obtained using untargeted ^1^H nuclear magnetic resonance (NMR) spectroscopy on four to eight gill tissue samples for each treatment. Metabolites were extracted using methanol–chloroform, subsequently dried overnight, then resuspended in D_2_O ready for profiling. The chemical shift of the NMR signals was used to assign the metabolites and quantify them based on the internal database and integration routine within Chenomx NMR suite 8.1 (Chenomx Inc., Canada). Further procedural details are described in Götze et al. (2020). BCAAs originate either from the diet or from endogenous protein degradation. At thermal limits, where feeding ceases, accumulation likely results from the degradation of intracellular proteins, and hence can be used as an indicator of protein catabolism.

#### Rate of apoptosis

Caspase-3 and -7 activities were investigated in gill tissue to quantify the rate of apoptosis using the Caspase-Glo^®^ 3/7 Assay Systems (Promega, G8090). A 50-fold volume of buffer (25 mmol l^−1^ HEPES, pH 7.5, 5 mmol l^−1^ MgCl_2_, 1 mmol l^−1^ EGTA, 5 µg ml^−1^ full protease inhibitor mix that did not target the caspases themselves; Sigma-Aldrich, P8340) was added to approximately 20 mg of gill tissue. Homogenization comprised two steps: first, samples were dispersed on ice with a Heidolph Silent Crusher (three times 15 s with 10 s on ice in between), and then by ultrasonication (two times 30 s at 4°C and 40% power, Branson Sonifier 450). Samples were centrifuged (15 min, 13,000 ***g***, 4°C) and equal volumes of supernatant and assay reagent were added and incubated for 1 h at room temperature in the dark. Luminescence was detected with a Fluoroskan Ascent FL microplate reader (Thermo Fisher Scientific). Relative values were corrected for the respective soluble protein content of each sample, which was detected via Bradford assay (Bradford, 1976; bovine serum albumin as standard) and expressed as relative luminescence units (RLU) per mg protein.

### Statistical analysis

Temperature-induced differences in filtration rate were detected by linear models accounting for repeated measures, either with the effect of temperature assumed to be linear, using regressions, or not linear, using ANOVA followed by *post hoc* pairwise comparisons using the Holm–Šidák method. Additionally, the temperature-dependent performance curve of filtration rate was fitted according to Schmalenbach et al. (2009):
(4)

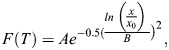
where *A*, *B* and *x*_0_ are coefficients of variation and *x* is the experimental temperature filtration rate.

We highlight discontinuities in the temperature dependent slopes of RMR using Arrhenius plots. The Arrhenius break temperature (ABT) was calculated from intersections of two linear regressions according to Nickerson et al. (1989) and Yeager and Ultsch (1989). However, the descending regressions were poorly constrained, both with few observations and few temperature steps, complicating estimation of ABT. Therefore, we use them for evidence whether ABTs are different between treatments, rather than precise values. Linear regressions followed the equations shown in the Fig. 2 legend.

Both temperature-induced and treatment-specific differences in haemolymph *P*_O_2__, mantle pH_i_, and branchial levels of MDA, HSP70, ubiquitin, BCAAs and caspase activity were detected by two-way ANOVAs, followed by *post hoc* pairwise comparisons, again using the Holm–Šidák method. Normality and/or homogeneity of variance was assessed by histograms and equal variance tests. If these linear model assumptions were violated, data were ln-transformed prior to analysis.

All analyses were two-tailed with the significance threshold *P*<0.05. All data are given as means±s.d. if not indicated otherwise. Statistical analyses were carried out either using Sigma Plot 12.0 (Systat Software) or, for the filtration rate and RMR analyses, R 4.3.0 (https://www.r-project.org/).

## RESULTS

### Whole-organism responses

#### Filtration rate

**Fig. 1.**
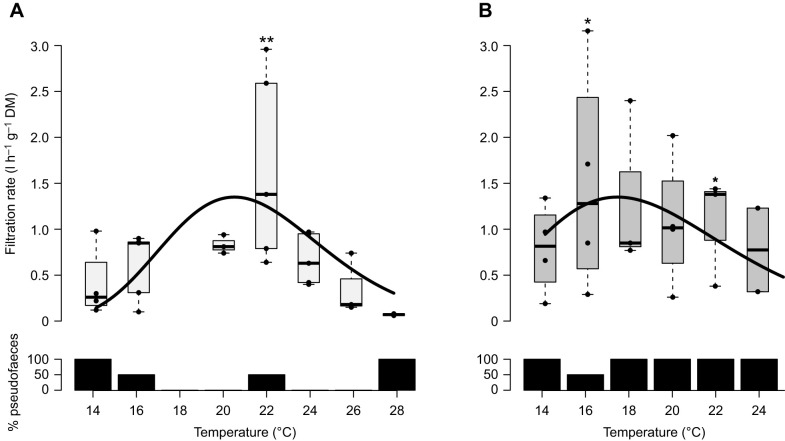
**Temperature-dependent curves of filtration rate and pseudofaeces ejection of *Pecten maximus* under warming-only (W) and deadly trio (DT) treatments.** (A) Warming under normocapnia and normoxia (W) leads to a performance curve maximum between 20°C and 24°C. (B) Deadly trio (DT; warming under hypercapnia: 1850 ppm and mild hypoxia: 55%) leads to a performance curve maximum between 16°C and 22°C. Asterisks denote significant filtration rate differences (*P<0.05, **P<0.001) of temperature steps relative to 14°C within each treatment (see Table S2A over both treatments). Box plots show the median (central line), interquartile range (box; IQR) and whiskers cover observations within 1.5 IQR. W treatment *N*=5, except *N*=4 for 14°C, *N*=3 for 20 and 26°C, *N*=2 for 28°C. DT treatment *N*=4 except for *N*=3 for 18 and 22°C, *N*=2 for 24°C. Non-linear regressions (black line) show the filtration rate modelled following Schmalenbach et al. (2009), described by the curves under (A) W, 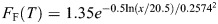, *r^2^*=0.31; and (B) DT, 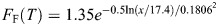, *r*^2^=0.08. Bar plots (bottom) depict observations of pseudofaeces ejection, where the remainder (%) consists of faeces, except after death of all scallops at 26°C under DT exposure.

Filtration rate of *P. maximus* rose from 0.74±0.97 l h^−1^ g^−1^ DM at 14°C in a temperature-dependent performance curve that peaked at 22°C with 1.80±0.67 l h^−1^ g^−1^ DM (pairwise comparison, *P*<0.001; Table S2A; Fig. 1). Although the filtration rate was not significantly affected by treatment (*P*=0.4; Table S2A), high filtration rates at 16°C under DT exposure helped make the increase in rates at this temperature marginally significant over those at 14°C (*P*=0.049; Table S2A). Beyond 24°C, all four individuals had ceased filtering under DT. This was the case for only two out of five individuals under W (Fig. 1B). Pseudofaeces ejection differed between W and DT exposures (Fig. 1), occurring predominantly at low (14–16°C) and high (28°C) temperatures during warming alone, whereas DT-exposed scallops produced pseudofaeces irrespective of temperature.

**Fig. 2.**
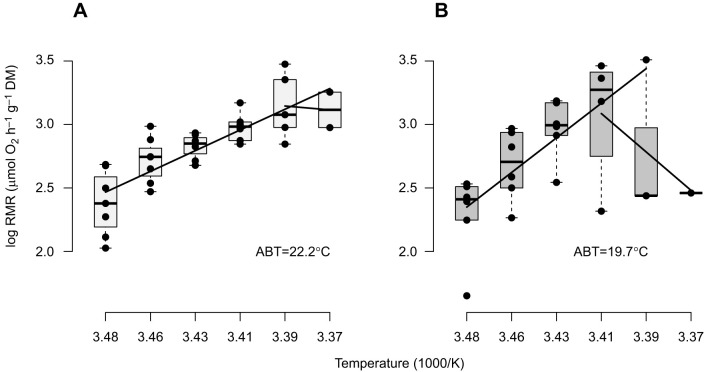
**Arrhenius plots showing the temperature dependence of *P. maximus* routine metabolic rate (RMR) under warming-only (W) and DT treatments.** (A) Warming under normocapnia and normoxia leads to an estimated Arrhenius break temperature (ABT) at 22.2°C with the ascending equation ln(RMR)=(−6.96×*T*)+26.71 between 14°C and 24°C (note the slope is negative with inverted temperature) and the descending equation ln(RMR)=(1.33×*T*)+−1.37 between 22°C and 24°C. (B) Deadly trio (warming under hypercapnia and hypoxia) led to an ABT at 19.7°C with the ascending equation ln(RMR)=(−11.55×*T*)+42.59 between 14°C and 20°C and the descending equation ln(RMR)=(13.18×*T*)+−41.88 between 20 and 24°C. Box plots show the median (central line), interquartile range (box; IQR) and whiskers cover observations within 1.5 IQR. W treatment *N*=7, except *N*=6 for 20°C, *N*=5 for 22°C, *N*=2 for 24°C. DT treatment *N*=6, except *N*=5 for 18°C, *N*=3 for 20 and 22°C, *N*=1 for 24°C. See Table S2B for statistics comparing the ascending slopes over both treatments. *x*-axis temperatures in °C, from left to right, are 14, 16, 18, 20, 22 and 24.

#### Routine metabolic rate

Under warming alone, RMR at 14°C was 11.1±2.8 µmol O_2_ h^−1^ g^−1^ DM (*N*=7) and increased to a maximum at 22°C of 23.9±6.3 µmol O_2_ h^−1^ g^−1^ DM (*N*=5), whence it levelled off (ABT=22.2°C; Fig. 2A). Under DT, RMR at 14°C was 10.3±2.7 µmol O_2_ h^−1^ g^−1^ DM (*N*=6), increased to a higher peak of 28.3±3.9 µmol O_2_ h^−1^ g^−1^ DM at 20°C (*N*=3) and dropped with further warming (ABT=19.7°C; Fig. 2B). A linear model of the ascending RMR Arrhenius curves, covering the shared temperatures of 14–20°C, suggested that the temperature-dependent increase in RMR, with mean *Q*_10,14–20°C_ values of 5.4 (DT) and 2.6 (W), was not significantly different between W- and DT-exposed scallops (*P*=0.4; Table S2B). The insignificance of difference between treatments likely followed the small sample size with repeated observations. Mortality within the animals labelled for RMR was at lower temperatures but otherwise reflected mortality in the wider experiment (detailed in Götze et al., 2020), starting around 20°C in DT-exposed scallops, and at 22°C in W-exposed scallops, with all animals dying during the transition to 26°C.

#### Haemolymph *P*_O_2__

**Fig. 3.**
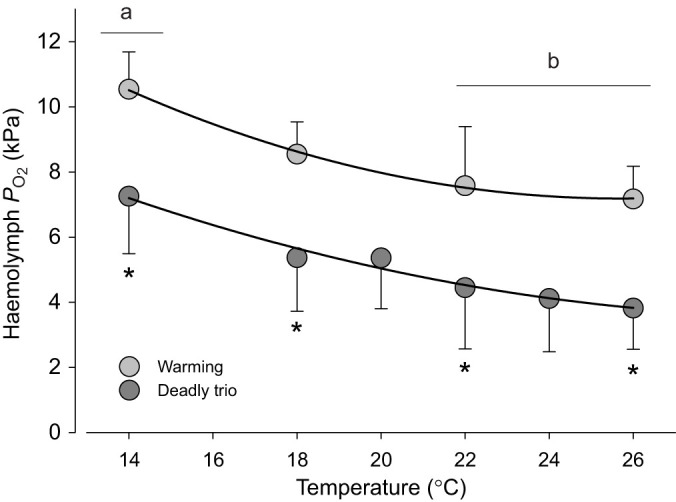
***Pecten maximus* haemolymph *P*_O_2__ decreases with temperature under warming-only (W) and deadly trio (DT) treatments**. *P*_O_2__ is significantly lower than under warming only. Data are means±s.d. W treatment *N*=7, except *N*=6 for 14°C, *N*=5 for 18°C. DT treatment *N*=6, except *N*=5 for 14°C and 18°C, *N*=4 for 22°C and 26°C. Statistics in Table S3A. The different letters indicate significant differences by temperature within one treatment (*P*<0.001). Asterisk indicates significant differences between treatments at the same temperature (pairwise comparisons, all *P<0.01).

Scallop haemolymph *P*_O_2__ was significantly lower under DT than W by a mean difference of ∼3.24 kPa (treatment effect, *P*<0.001; Fig. 3, Table S3A). Progressive warming caused a significant decrease in haemolymph *P*_O_2__ (*P*<0.001) from 10.5±1.2 kPa at 14°C to 7.2±1.0 kPa at 26°C in W-exposed scallops, and from 7.3±1.8 to 3.8±1.3 kPa under DT (pairwise comparisons, both *P*<0.001). The rate of decrease was not significantly different between treatments (*P*=0.998; Table S3A).

### Cellular responses

#### Intracellular pH

**Fig. 4.**
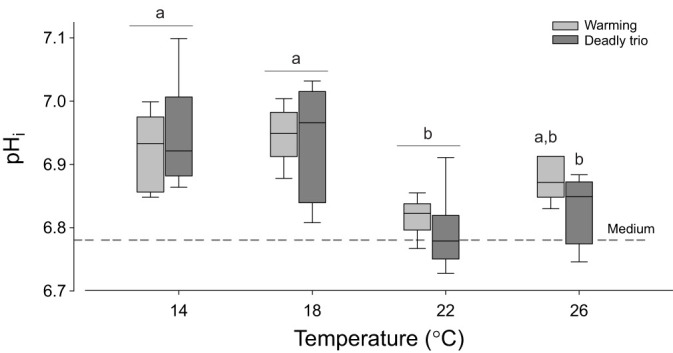
***Pecten maximus* mantle tissue intracellular pH (pH_i_) decreased with temperature but was not different between warming-only (W) or deadly trio (DT) treatments**. Statistics in Table S3B. Box plots show the median (central line), interquartile range (box; IQR) and whiskers cover observations within 1.5 IQR. *N*=6 except *N*=5 for W-treatment 18°C and for DT treatment 18°C and 26°C. The different letters indicate significant differences by temperature within one treatment (pairwise comparisons, *P*<0.01).

Cumulative warming significantly decreased mantle pH_i_ in both groups (*P*<0.001; Fig. 4; Table S3B) but the pH_i_ values of the two treatments were not significantly different to one another. pH_i_ was significantly lower at 22°C than at 14–18°C (Holm–Šidák pairwise comparisons, both *P*<0.03 for W; both *P*<0.03 for DT). Assuming a linear relationship, for simplicity, between temperature and pH_i_ would give a mean decrease in pH_i_ per degree Celsius of 0.007 pH units (95% CIs=0.012–0.001, *P*=0.02, warming-only treatments; Table S2C; or including DT observations, conditional mean=0.009, 95% CIs=0.014–0.005, *P*=0.0002; Table S2D).

#### Lipid peroxidation

**Fig. 5.**
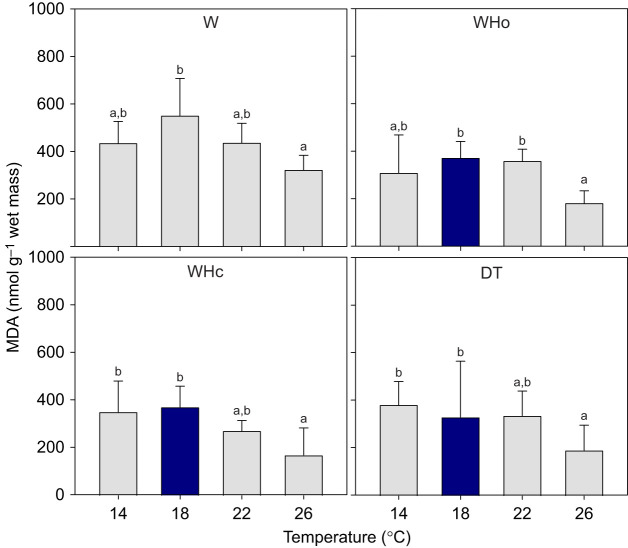
***Pecten maximus* gill tissue malondialdehyde (MDA) was lower at high temperatures under warming-only (W), warming plus hypoxia (WHo), warming plus hypercapnia (WHc) and deadly trio (DT) treatments**. MDA was also lower at 18°C in the combined stressor treatments than in the warming-only treatment. Shown are means±s.d. *N*=6, except W treatment *N*=5 for 14°C, *N*=4 for 18°C, Who treatment *N*=4 for 26°C, WHc treatment *N*=7 for 26°C, DT treatment *N*=5 for 18°C and 26°C. Statistics in Table S3C. The different letters indicate significant changes with temperature within one treatment (pairwise comparisons *P*<0.05; Table S3C). Dark blue bars indicate significant differences between the respective value and its equivalent under the warming-only treatment (pairwise comparisons, *P*<0.02).

Warming significantly decreased MDA levels in gills of *P. maximus* (*P*<0.001; Fig. 5) irrespective of whether additional stressors were present (interaction term, *P*=0.64). Mean values remained generally lower in the combined stressor treatments than with warming-only (*P*<0.001; Table S3C). Only at 18°C did additional stressors significantly decrease MDA levels relative to the warming-only treatment (pairwise comparisons, *P*<0.02), but this trend was visible at all other temperatures (Fig. 5).

#### HSP70

**Fig. 6.**
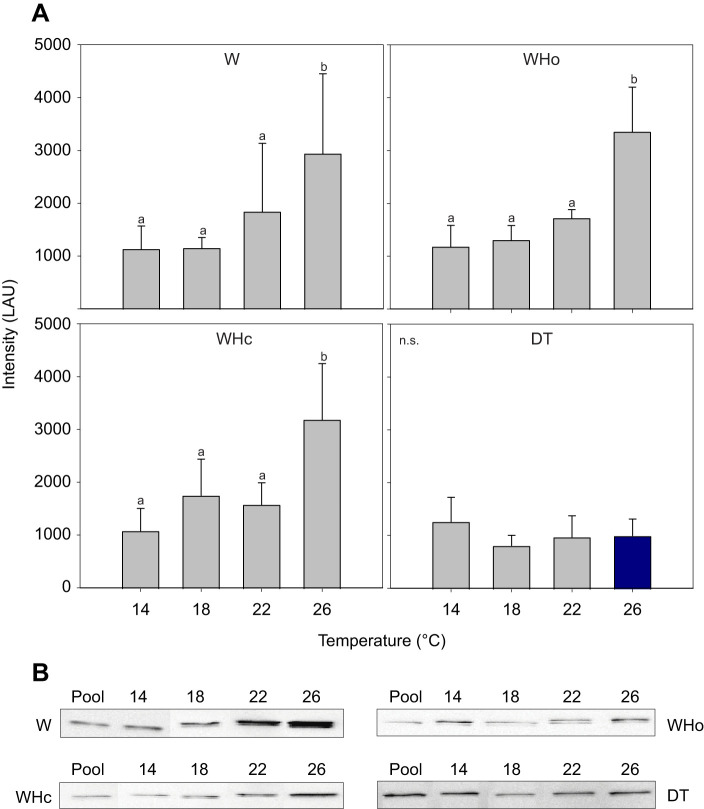
**Generally increasing heat shock protein 70 content (HSP70) in *P. maximus* gill tissue with temperature under warming-only (W), warming plus hypoxia (WHo) and warming plus hypercapnia (WHc) treatments, but not in deadly trio (DT) treatment.** (A) Normalized levels of HSP70 given in luminescence arbitrary units (LAU). Shown are means±s.d. *N*=6, except W treatment *N*=5 for 18°C and 22°C, Who treatment *N*=5 for 14 and 18°C, *N*=4 for 26°C, WHc treatment *N*=5 for 14°C, DT treatment *N*=5 for 18°C, *N*=4 for 26°C. Statistics in Table S3D. Letters indicate significant changes with temperature within one treatment (higher only at 26°C, *P*<0.001). Dark blue bar indicates significantly lower HSP70 only at 26°C under DT than at 26°C under warming-only (*P*<0.001). (B) Exemplary immunoblot images of temperature dependent HSP70 signals for each treatment, omitting the faint second band.

Temperature increased scallop branchial HSP70 levels in W, WHo and WHc treatments alike (*P*<0.001) but not in DT-exposed scallops, leading to a significant interaction term (*P*=0.012; Table S3D; Fig. 6). The effect of warming on HSP70 was significant in W, WHo and WHc treatments only once 26°C was reached (*P*<0.001). In contrast, warming had no significant effect on DT-exposed scallop HSP70, which remained low irrespective of temperature, being significantly lower at 26°C than in the W-exposed control (pairwise comparison, *P*<0.001).

#### Ubiquitin conjugates and proteinogenic BCAAs

**Fig. 7.**
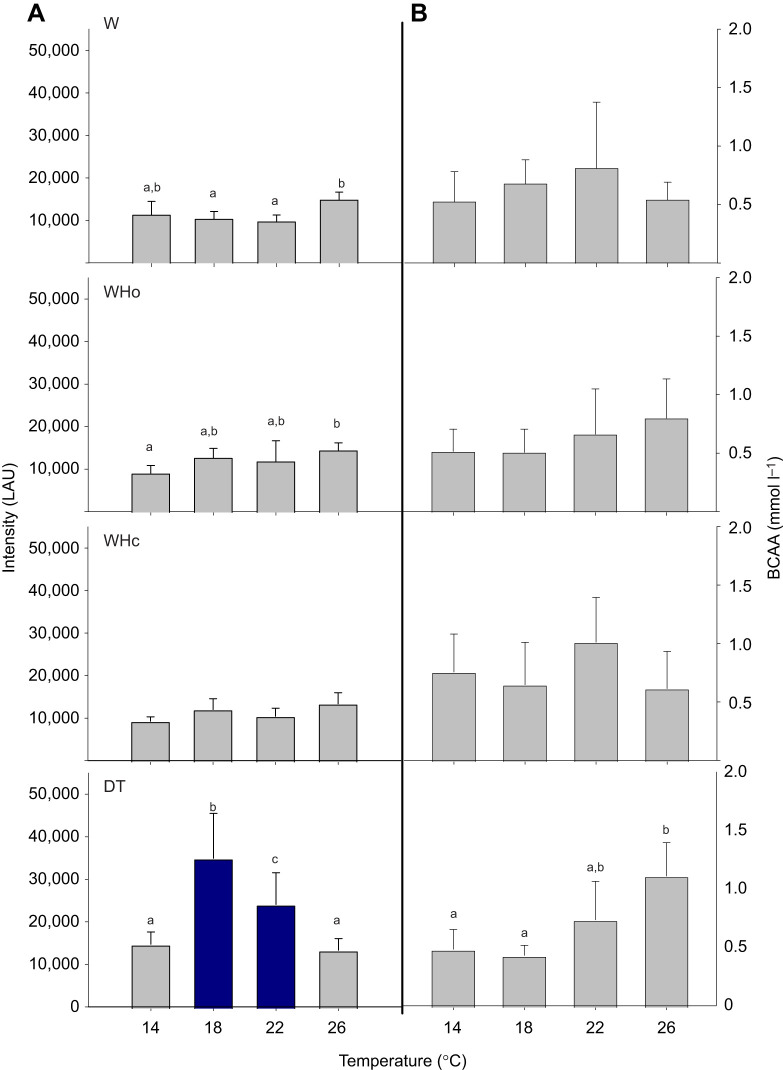
***Pecten maximus* gill tissue ubiquitin and branched chain amino acids (BCAAs) under warming-only (W), warming plus hypoxia (WHo), no change under warming plus hypercapnia (WHc), and substantial changes under deadly trio (DT) treatments**. Shown are means±s.d. (A) Normalized levels of ubiquitin conjugates given in luminescence arbitrary units (LAU) were temperature dependent (*P*<0.001), with significant changes with temperature within one treatment (letters, pairwise comparisons *P*<0.05). Dark blue bars indicate significantly higher ubiquitin content relative to under W treatment (pairwise comparisons all *P*<0.001). *N*=6, except W treatment *N*=5 for 22°C, *N*=7 for 26°C, Who treatment *N*=5 for 14°C, *N*=4 for 26°C, WHc treatment *N*=5 for 14°C, DT treatment *N*=5 for 18 and 26°C. (B) BCAA concentrations as the summed levels of the cytosolic amino acids leucine (Leu), isoleucine (Ile) and valine (Val) were weakly temperature dependent (*P*=0.034, individual concentrations adopted from Götze et al., 2020). Significant changes within a treatment only observed for DT (letters, pairwise comparisons *P*<0.02). *N*=6, except W treatment *N*=5 for 18°C and 22°C, *N*=7 for 26°C, Who treatment *N*=5 for 22°C, *N*=4 for 26°C, WHc treatment *N*=8 for 26°C, DT treatment *N*=5 for 18°C and 26°C. Statistics in Table S3E,F.

The effect of temperature on normalized levels of branchial ubiquitin conjugates depended on treatment (Fig. 7A) (interaction term, *P*<0.001; Table S3E). W- and WHo-exposed scallops showed a small but significant accumulation in ubiquitin conjugates at 26°C compared with 18°C and 22°C (W group, *P*<0.018) or with 14°C (Who group, *P*=0.010), whereas the accumulation in WHc-exposed scallops was only marginally significant (14°C versus 26°C, *P*=0.054). In contrast, scallops that were DT-exposed had significantly higher levels of ubiquitinated proteins at 18°C and 22°C compared with both 14°C and 26°C (*P*<0.001). These levels were also significantly higher than in scallops at equivalent temperatures in the W treatment (pairwise comparisons, both *P*<0.001).

Branchial BCAA concentrations were overall correlated with temperature (*P*=0.034). However, the only clear trend was in the DT treatment, where BCAA levels were significantly higher at 26°C than at 14°C and 18°C (pairwise comparisons, *P*<0.02; Table S3F; Fig. 7B). This led to a marginally significant increase in BCAA levels in DT-exposed over W-exposed scallops at 26°C (*P*=0.052).

#### Oxygen radical absorbance capacity

**Fig. 8.**
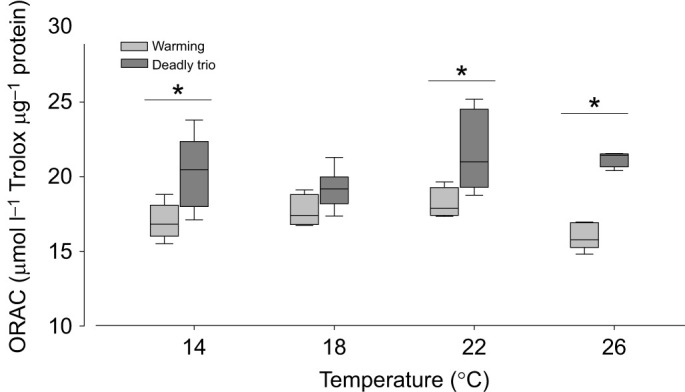
***Pecten maximus* gill tissue oxygen radical absorbance capacity (ORAC) increased with temperature in scallops of the deadly trio (DT) but not the warming-only (W) treatment**. *N*=5, except W treatment *N*=4 for 18°C and 22°C, DT treatment *N*=6 for 18°C, *N*=4 for 26°C. Asterisk indicates significant differences between treatments at the same temperature (*P<0.05; statistics in Table S3G).

Gill tissue ORAC was significantly higher in DT-exposed than W-exposed scallops (*P*<0.001; Fig. 8; Table S3G), specifically at 14°C, 22°C and 26°C (each *P*<0.01), but not at 18°C.

#### Rate of apoptosis

**Fig. 9.**
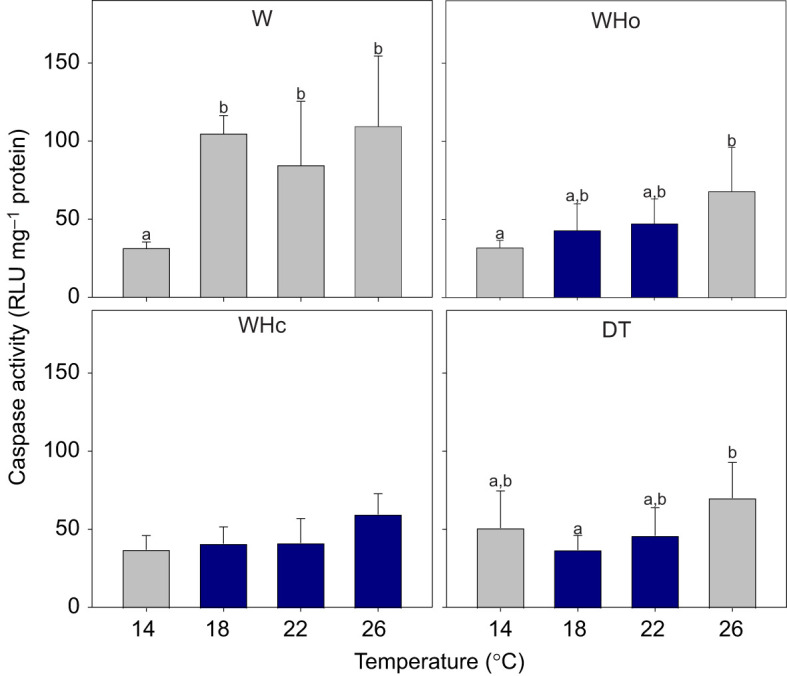
***Pecten maximus* gill tissue activity of caspase-3 and -7 was increased at higher temperatures under warming-only (W) and warming plus hypoxia (WHo), but not clearly under warming plus hypercapnia (WHc) or deadly trio (DT) treatments**. Multiple stressor treatments show significantly lower activity than the W treatment. Shown are means±s.d. *N*=6, except W treatment *N*=5 for 18°C, Who treatment *N*=4 for 26°C, WHc treatment *N*=5 for 26°C, DT-treatment *N*=5 for 26°C. Statistics in Table S3H. Different letters indicate significant changes with temperature within one treatment (pairwise comparisons *P*<0.01). Dark blue bars indicate significant differences to the respective temperature of the W treatment (pairwise comparisons, *P*<0.04). RLU, relative luminescence units.

The activity of branchial caspase-3 and -7 increased significantly with temperature (*P*<0.001; Table S3H) though varied with treatment (interaction term, *P*<0.001; Fig. 9). Although warming strongly increased caspase activity in W-exposed scallops, it had only a marginally significant effect in WHc-exposed scallops (26°C versus 14°C, *P*=0.09), and significant but less strong effects in the WHo- (26°C versus 14°C, *P*=0.007) and DT-exposed scallops (significant only from 26°C versus 18°C, *P*=0.01). Compared with the same temperatures in the warming-alone treatment, caspase activity was significantly lower in WHo-, WHc- and DT-exposed scallops at 18°C and 22°C (pairwise comparisons, maximum *P*=0.03), and in WHc-exposed scallops at 26°C (pairwise comparison, *P*=0.03).

## DISCUSSION

Climate change impacts marine ectotherms at multiple levels of biological organization (e.g. reviewed by Pörtner, 2002; Kassahn et al., 2009; Sokolova et al., 2012; Pörtner et al., 2017), with warming, hypoxia and hypercapnia often interacting to threaten organisms and marine ecosystems (Pörtner et al., 2014; IPCC, 2019). This ‘deadly trio’ was hypothesized to contribute to ancient mass extinctions (Bijma et al., 2013; Kiessling et al., 2024), such that unifying mechanisms may underlie both ancient extinction responses and those observed under current climate-related stress (Reddin et al., 2020), as unravelled by experimental research (Pörtner et al., 2004; Pörtner and Knust, 2007; Pörtner and Farrell, 2008; Pörtner, 2010; Calosi et al., 2019). The present study aimed to elucidate how hypoxia and/or hypercapnia modify responses to cumulative warming exposure in a scallop, the warming-only ramp serving as a control, covering the same experimental temperatures and durations. Thereby, we aimed to reveal the mechanisms limiting whole-organism performance and further elucidate those shaping the concept of OCLTT (Pörtner, 2002, 2010; Lannig et al., 2010; Zittier et al., 2015; Stapp et al., 2017; Eymann et al., 2020; Matoo et al., 2021). Our present data are in line with earlier findings that changes in cardio-ventilatory parameters, oxygen consumption (*Ṁ*_O_2__) and haemolymph *P*_O_2__ characterize the onset of temperature-induced impairments in marine organisms (Frederich and Pörtner, 2000; Artigaud et al., 2014; Tripp-Valdez et al., 2017). In suspension-feeding bivalves, oxygen uptake, feeding and excretion of nitrogenous wastes co-occur during active ventilation. Therefore, temperature-dependent ventilation volume, which enables the rate of filtration, is a major indicator of the aerobic power budget (Guderley and Pörtner, 2010) and as such is considerably affected by water viscosity and physiological responses (Møhlenberg and Riisgård, 1978; Jørgensen et al., 1990; Specht and Fuchs, 2018; Eymann et al., 2020). Cellular responses are more complex and context dependent, but in sum would underpin the whole-organism responses seen. For example, our results suggest that routine apoptosis accompanies warming into and beyond thermal optima as cells become increasingly stressed and damaged, whereas DT allows cellular damage to escalate by preventing that apoptosis.

In cumulative heat ramp experiments, such as deployed here, exposure duration above thermal optima may contribute to stress and, depending on the species, may elicit different tolerance mechanisms (e.g. Götze et al., 2025 for *O. edulis*; see Materials and Methods for further discussion). Here, thermal optimum was between 18°C and 22°C, based on peak filtration rate, such that tolerance likely became time-limited at 24°C, marking 10 days since the treatment began but 2 days above thermal optima, especially with additional stressors. Thus, 26°C represents 4 days and 28°C represents 6 days above optimum.

### Whole-organism responses

Routine metabolic rate (RMR) of *P. maximus* increased progressively with warming, reaching its maximum and leaving the optimum range at 22°C, in line with findings by Artigaud et al. (2014). Several studies have demonstrated similar temperature-dependent RMR curves in marine molluscs (e.g. Bougrier et al., 1995; Haure et al., 1998; Tripp-Valdez et al., 2017). The warming-induced decrease in *P*_O_2__ alongside increasing metabolic rates in various bivalve species (*P. maximus* in Schalkhausser et al., 2014, and the present study; oyster *Crassostrea gigas* in Lannig et al., 2010) indicates a progressive mismatch between oxygen demand and oxygen supply capacities, which can finally lead to an onset of anaerobic metabolism. In our parallel study on *P. maximus* (Götze et al., 2020), we found a warming-induced accumulation of acetate by 26°C. A transition to anaerobiosis by 26°C and our observation of RMR levelling off at 24°C, despite progressive warming, indicate that the increasing energy demand is no longer fully covered by aerobic energy generation. According to Pörtner (2001), the temperature-induced onset of anaerobiosis marks a critical temperature, which is related here to the temperature at which 50% of the animals died (LT_50_) at ∼25.0°C (Götze et al., 2020). LT_50_ tends to be close to the ABT in heart rate or oxygen consumption (Stillman and Somero, 2000; Pörtner et al., 2017) although their correspondence may depend on the rate of warming (Götze et al., 2025). We conclude that under gradual warming, without additional stressors, *P. maximus* entered its critical thermal range between 25°C and 26°C.

Whole-organism responses to DT imply a downward shift of upper thermal limits in adult *P. maximus* (Schalkhausser et al., 2013), associated with a stronger stimulation of RMR as indicated by a higher DT *Q*_10,14–20°C_ of 5.4 compared with 2.6 under normocapnic and normoxic warming. Meanwhile, a lower RMR ABT around 20°C, rather than around 22°C, likely indicates reduced capacity of oxygen supply to meet demand under hypercapnia, in line with a depression of motile function during hypercapnic acidosis (Wittmann and Pörtner, 2013). Although the temperature-dependent decrease in *P*_O_2__ was similar in DT- and W-exposed scallops, *P*_O_2__ remained at significantly lower values in DT-exposed scallops. It was unclear whether the values of lower DT *P*_O_2__ were because of lower *P*_O_2__ in the DT-treatment water or additionally because of perturbations resulting from additional hypercapnia. This mirrors a transition towards anaerobic energy production at lower critical temperatures, evidenced by a significant accumulation of acetate already at 18°C and 22°C, followed by succinate accumulation at 26°C, in DT-exposed scallops (Götze et al., 2020), as soon as haemolymph *P*_O_2__ fell below 6 kPa (see also Schalkhausser et al., 2013). A lowered LT_50_ of 22.5°C in DT-exposed scallops, relative to 25°C in W-exposed scallops, also supported that scallops surpassed the critical temperature (*T*_crit_) at lower temperatures in DT than in warming alone (Götze et al., 2020). Similarly, *Haliotis flugens* experienced a clear downward shift of thermal tolerance limits under DT, associated with a rise in *Q*_10_ from 1.72 to 2.21 (Tripp-Valdez et al., 2017).

Populations of *P. maximus* from northwestern Spain (Galicia) in 1990 experienced seasonal temperature variation spanning at least 11°C to 21°C (data averaged over 4–10 m depth from a raft; Pazos et al., 1997), the upper end of which would cover our observed peak in filtration performance, between 18°C and 22°C. Filtration rate-derived performance curves of the sympatric intertidal species *Mytilus edulis* and *Ostrea edulis* (Schulte, 1975; Eymann et al., 2020) showed filtration rates were maintained to higher temperatures, to 25°C and 30°C, respectively. Importantly, *O. edulis* specimens were warmed at the same rate (Eymann et al., 2020) as the *P. maximus* here, suggesting *O. edulis* to have greater thermal tolerance. DT exposure of *P. maximus* made the thermal optimum of filtration indistinct, as also evident in pseudofaeces production, indicating the influence of combined drivers severely constrained active tolerance.

**Fig. 10.**
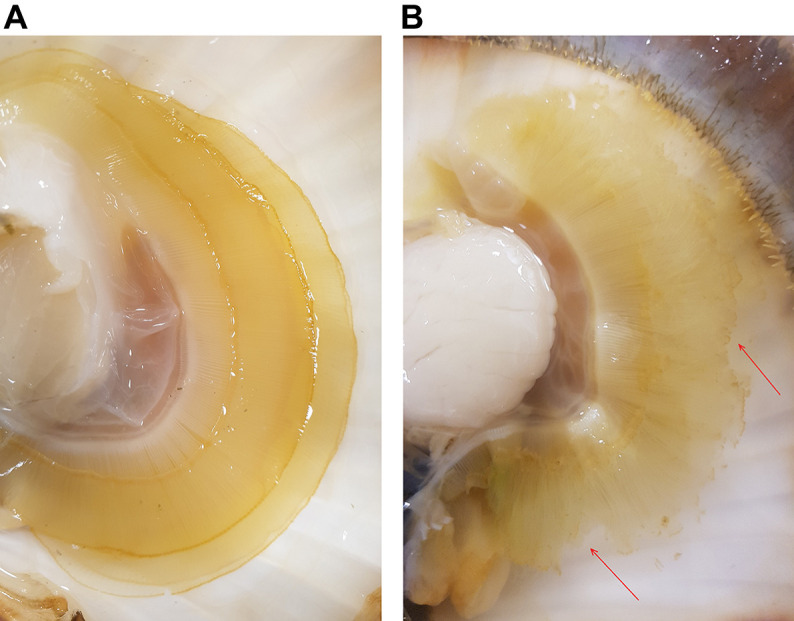
**Example photographs of *P. maximus* gill condition taken during sampling.** (A) Gills of control scallops maintained at 14°C (warming). (B) Gills of *P. maximus* exposed to 26°C and DT. Red arrows highlight areas with visible gill destruction. No clear gill destruction was visible in any of the other exposures (W, WHo or WHc) at 26°C but, unfortunately, no control photos were taken for this unexpected result, with DT exposure being the last exposure to be completed.

Bivalves regulate food uptake, rejecting undesired material as pseudofaeces, while actively selecting food items according to particle size and nutrient content as a result of algal fitness (Møhlenberg and Riisgård, 1978; Laing and Psimopoulous, 1998; Brilliant and MacDonald, 2002). Here, *P. maximus* pseudofaeces production occurred mainly at the lowest (14°C) and highest (26°C) temperatures when exposed to warming alone, but not in between, making filtration inhibition owing to algal oversaturation or disturbance unlikely. Algal cultures were checked daily for their fitness, so we also doubt increased pseudofaeces discharge owing to unpreferred dead material to be the reason. We hypothesize that pseudofaeces production in the warming-only treatment followed particles being rejected intentionally to avoid digestive costs, reducing ATP demand during the transition towards anaerobiosis at both thermal extremes. Similarly, DT-exposed scallops, which produced pseudofaeces throughout, may have faced constraints over the full thermal range in line with the reduced haemolymph *P*_O_2__. A potential mechanism may involve insufficient ATP generation limiting energy-dependent cilial strokes (Clemmsen and Jørgensen, 1987) and leading to faulty particle transport via the gills to the digestive tract and to earlier deprioritization of feeding. Furthermore, degradation of gill tissue at elevated temperature under DT (Fig. 10, see below), but not under warming alone, indicated loss of gill morphology used for particle trapping, which may also constrain food consumption (Beninger and St-Jean, 1997; Beninger et al., 1999). Because food and oxygen uptake are linked in bivalve filter feeding, the loss of exchange surface area in the gills may also result in decreased oxygen availability to tissue, triggering a downward shift of upper critical temperature and associated transition to anaerobic metabolism and energy deficiency. We conclude that DT elicited a severe narrowing of the thermal window of active tolerance, hastening passive time-limited survival. A strengthened warming-induced mismatch between oxygen supply and demand under DT is corroborated by the decreasing haemolymph *P*_O_2__ with increasing temperature (Fig. 3).

*Pecten maximus* responds to rising temperature as an oxy-regulator (Artigaud et al., 2014), where the warming-induced exponential increase in metabolic rate causes the critical level for *P*_O_2__ to rise with increasing temperature (Brown et al., 2004; Vaquer-Sunyer and Duarte, 2011). In Artigaud et al. (2014), *P. maximus* critical *P*_O_2__ rose from 1.7 mg O_2_ l^−1^ at 10°C to 2.4 mg O_2_ l^−1^ at 25°C. Similar findings were obtained in mussels by Jansen et al. (2009), with critical *P*_O_2__ shifting upward to normoxic oxygen levels, reflecting the characteristics of *T*_crit_ in OCLTT. In the present DT exposure, the lowest water *P*_O_2__ of 3.4±0.3 mg l^−1^ at 26°C was still higher than *P. maximus* critical *P*_O_2__ at 25°C warming (Table S1). This suggests that the oxygen deficiency introduced under DT may lead to a prioritization of gaseous exchange over feeding, causing enhanced pseudofaeces production (see above) and, finally, mortality at lower temperatures. DT exposure reduced LT_50_ from 24°C under both WHo or WHc, or from 25°C under warming alone, to 22.5°C (Götze et al., 2020). Overall, such narrowing of the thermal niche under DT and constraint on aerobic performance optimum likely resulted from an imbalance between oxygen demand and supply, involving a steep increase in demand as reflected in the high *Q*_10_ value at already reduced haemolymph *P*_O_2__, alongside a lack of inducible HSP70 response but a much stronger and earlier induction of ubiquitin-conjugated proteins (see below).

Warming plus hypercapnia elicits a strong response of acid–base regulation in marine organisms (Pörtner et al., 2004; Wittmann and Pörtner, 2013). Marine bivalves are expected to be particularly vulnerable to hypercapnia as they possess low capacities for acid–base regulation (e.g. Melzner et al., 2009; Sokolova et al., 2024). However, data from the literature suggest that *P. maximus* might be tolerant to hypercapnia exposure alone (Burton Sanders et al., 2013; Cameron et al., 2019), perhaps because of its lifestyle with relatively intense bursts of activity (i.e. escape via swimming). For example, 3 months of exposure to a CO_2_ partial pressure of 1140 μatm had no impact on feeding, breathing, cellular turnover or condition index (Burton Sanders et al., 2013). Schalkhausser et al. (2014) found unchanged oxygen consumption rates and only a marginal reduction of escape performance of adult *P. maximus* under warming plus hypercapnia (1120 μatm *P*_CO_2__ at either 10°C or 20°C for 50 days). *In vivo* muscle bioenergetics showed a progressive decrease in resting levels of phospho-L-arginine owing to warming (15°C versus 20°C) and warming plus hypercapnia (1120 μatm *P*_CO_2__; Bock et al., 2019). Such lowering of energy reserves and adjustments of energy-related pathways (Bock et al., 2024) would explain their observed decrease in escape performance in moderate warming plus hypercapnia. However, warming and oxygen reduction impacts *P. maximus* more severely (Götze et al., 2020; see below), particularly if all three drivers are combined.

### Cellular responses

#### Within the aerobic performance range

Between 14°C and 22°C, we found neither oxidative stress, macromolecular damage nor induction of HSP70 in *P. maximus*. The present study even revealed decreasing branchial levels of MDA, an indicator for lipid peroxidation due to oxidative stress, at the highest temperature regardless of exposure to additional stressors. Bivalve studies that investigated oxidative damage of lipids in response to climate stressors either reported an induction of oxidative stress, and hence increased levels of damaged lipids (e.g. Woo et al., 2013; Sui et al., 2017; Liao et al., 2019), or constant MDA levels (e.g. Sussarellu et al., 2012; Matoo et al., 2013). On the low temperature side of the performance curve, the present findings of lowered MDA upon warming may reflect a warming-induced improvement of antioxidative defence effectiveness, despite oxygen radical absorbance capacity remaining constant (Fig. 8).

Balanced whole-organism and cellular homeostasis is likely restricted to within the aerobic thermal performance range. Therefore, elevated activities of caspase-3 and -7 beyond 18°C (Fig. 9), indicating an upscaling of apoptosis, may appear surprising. Apoptosis in marine bivalve mitochondria follows the intrinsic apoptotic pathway (Romero et al., 2015), and is usually observed after cell stress and damage, whereas premature activation of programmed cell death can be prevented, e.g. by anti-apoptotic mechanisms (e.g. Riedl and Shi, 2004). For instance, in the mussels *Mytilus galloprovincialis* and *M. californianus*, acute heat stress caused significant breaks in DNA strands in haemocytes, followed by caspase-3 activation (Yao and Somero, 2012). In *Crassostrea virginica*, similar heat stress caused oxidative stress and increased rates of apoptosis in gills and digestive gland tissues (Rahman and Rahman, 2021). The increase in caspase-3/-7 activity observed within the aerobic window may alternatively reflect adjustments in steady-state functioning to meet higher rates of cellular turnover with warming up to 22°C. Strahl and Abele (2010) suggested that scallops minimize cellular stress and damage by enhanced cell turnover rather than by extended cellular defence and repair. They compared cell proliferation, apoptosis rates and antioxidant defence mechanisms between *Aequipecten opercularis* (Pectinidae), which is capable of short bursts of swimming, and *Arctica islandica* (Arcticidae), which is long-lived and much less active. *Aequipecten opercularis* had lower capacities of antioxidant defence relative to *A. islandica* but counterbalanced this through higher rates of cell proliferation and apoptosis. Energetic costs of this trait might be covered by scallops' capability to maintain higher standard metabolic rates and higher levels of mantle cavity *P*_O_2__ compared with other bivalves (Abele et al., 2010). However, such a strategy might shorten passive tolerance periods in more active compared with less active and/or long-lived bivalve species. This would apparently contradict expectations of population decline if extrapolated from the results of Peck et al. (2009), such as done for extinctions in the fossil record (Clapham, 2017). Peck et al. (2009) found more active species to survive to higher temperatures during warming of 1°C day^−1^ than sessile or less active species, albeit among a limited pool of 14 Antarctic species wherein activity rate and maximum body size were negatively correlated. According to OCLTT and present findings, a larger aerobic scope and smaller body size would support wider active thermal ranges, which might be beneficial in the short term, but at the expense of passive thermal ranges. However, comparing extinction risk of more active relative to less active organisms, specifically during ancient events of (long-term) warming and DT, instead supports enhanced risk of active organisms (Reddin et al., 2021). This research direction clearly needs further scrutiny, as it suggests a crucial role for passive thermal tolerance (e.g. Liao et al., 2021).

#### At and beyond thermal limits

Warming to 26°C revealed a clear upregulation of HSP70, indicating the onset of branchial heat stress beyond the critical temperature, when survival became strictly time limited. This indicates the scallops at 26°C to be well within the passive range of thermal tolerance (Pörtner, 2010, 2017). Artigaud et al. (2015) reported warming-induced upregulation of three HSP genes (HSP40, HSP70 and HSP90) in *P. maximus*, where HSP expression remained low below 21°C but was upregulated at 25°C. Tomanek (2008) suggested that subtidal bivalves, such as *P. maximus*, which live in stable environmental conditions compared with intertidal species, show warming-induced induction of HSPs only at temperatures beyond those regularly experienced in their habitat. Summer mean maximal sea surface temperatures near Vigo, Spain, are around 21°C (www.seatemperature.org) and, up to this temperature, HSP70 was not induced in our lab experiments, in line with the scallop's submerged, fully aerobic mode of existence.

#### Warming under additional stressor exposure

Under warming and hypoxia (WHo), the decline in ambient oxygen would cause a decline in haemolymph oxygen levels (here, the data supporting this derive from the DT treatment). Oxygen deficiency owing to ambient oxygen decline or increased demand at limited supply leads to a narrowing thermal range through downward shifts in upper (and upward shifts in lower) critical temperatures (Pörtner and Lannig, 2009; Pörtner, 2010). Upon warming under hypoxia (WHo), we observed branchial anaerobiosis and mobilization of lipid energy reserves through β-oxidation at a lowered upper critical temperature of 18°C (Götze et al., 2020). Both organismic and metabolite data showed that *P. maximus* suffered more under WHo than under WHc, visible as higher mortality at the highest temperature of the exposure (26°C), with only four scallops surviving in the WHo-exposed group compared with 17 specimens in the WHc-exposed group (Götze et al., 2020). However, the present study revealed no additional oxidative stress or macromolecular damage in gill tissue of WHo-exposed scallops (relative to warming only), indicating that the cellular stress response was not exacerbated by the level of ambient or systemic hypoxia. The induction of HSP70 in the WHo group began at 26°C, similar to findings in W-exposed scallops, and had a similar intensity (Fig. 6). However, in a study using immature *Argopecten purpuratus* (Brokordt et al., 2015), additional hypoxia doubled the levels of HSP70 compared with scallops exposed to thermal stress only, whereas this compounding effect of hypoxia was not seen in mature and spawned scallops. Some metabolic downregulation may have occurred in the latter, involving a downregulation of energy-demanding processes at lower levels of protection by HSP70 expression and related tolerance. Although the branchial HSP70 response was similar between W- and WHo-exposed scallops in our experiments, caspase­-3 and -7 activity increased in W-exposed scallops and was also somewhat lower in WHo-exposed scallops, suggesting that apoptosis as an energy-consuming process was also somewhat constrained by hypoxic exposure.

Hypercapnia on top of warming had only marginal effects on branchial metabolic pathways and survival (Götze et al., 2020; Bock et al., 2024), and was the only treatment where no significant difference in ubiquitin or caspase activity was observed between temperatures. Therefore, both branchial metabolism and cellular stress indicate that *P. maximus* is resilient to combined warming and short-term hypercapnia, although incurring negative impacts on swimming capability (Bock et al., 2019, 2024). Sun et al. (2025) also observed lower apoptosis indicators in *Mytilus coruscus* for combined warming and hypercapnia relative to warming alone.

Lastly, the combination of all three drivers, warming, hypercapnia and hypoxia (DT), can exacerbate the impacts of individual drivers (i.e. interacting effects), strongly modulating a species' thermal window and response, as discussed above and previously for abalone (Tripp-Valdez et al., 2017). In *P. maximus*, DT exposure causes an accumulation of glycogenic amino acids and anaerobic end-products of mitochondrial energy metabolism at 22°C and 26°C (Götze et al., 2020). The present data additionally revealed significant disturbances in branchial protein homeostasis under DT, visible as a destruction of gill tissue in DT-exposed scallops at 26°C (Fig. 10). More severe gill tissue histopathological changes also occurred in *M. galloprovincialis* subjected to combined hypoxia and hypercapnia, but not when the mussels were exposed to hypercapnia alone (Gostyukhina et al., 2024). The accumulation of damaged proteins in DT-exposed *P. maximus* at 18°C and 22°C was followed by increased levels of free BCAAs at 26°C, by which temperature the damaged (ubiquinated) proteins were back to 14°C levels. We hypothesize that the rise in BCAAs reflects hydrolysis of damaged proteins that had accumulated at 18°C and 22°C. High levels of damaged and ubiquinated proteins degrade cellular homeostasis, cause stress at the endoplasmic reticulum, and potentially induce apoptosis, necrosis and autophagy (see Orlowski, 1999; D'Arcy, 2019). However, despite the damage to cellular proteins, the rate of apoptosis was not elevated at any temperature in DT-exposed scallops, suggesting that apoptosis did not cause gill destruction. There was also no induction of HSP70, even though it is known that inhibition of the proteasome and accumulation of ubiquinated proteins trigger the induction of the heat shock response (Meriin et al., 1998; Young and Heikkila, 2010). At the same time, ORAC was increased under DT compared with warming at the highest temperature, whereas MDA levels decreased with temperature, which clearly did not compensate for the overall harmful effects of DT. Overall, these trends under DT constrain heat tolerance limits and time limitation to thermal tolerance. Further research is needed on the mechanisms underlying the exacerbating effects of DT. We hypothesize that DT strongly curtails acclimation capacity and thereby shortens the tolerance period that can be sustained under thermal stress. Low oxygen conditions and the exacerbated acidosis (Figs 3 and 4) together may elicit a strong depression of functionality and key protection mechanisms, in line with similar findings in abalone (Tripp-Valdez et al., 2017).

### Conclusions

We interpret our findings to indicate that *P. maximus* occupying the shallow subtidal near Vigo, Spain, may approach their upper thermal limit. Further warming and heatwaves, especially if combined with limited oxygen supply and elevated CO_2_, would elicit and exacerbate thermal stress and constraints in performance such that current shallow habitat may be lost, causing a shift to deeper waters or cooler areas, as seen across taxa (e.g. Poloczanska et al., 2013; Koenigstein et al., 2016). The interplay of warming and hypoxia induces a downward shift of critical temperature through an onset of anaerobiosis. However, warming in combination with both hypoxia and hypercapnia (DT) causes an even stronger impact through disturbance of cellular protective responses and reduced tolerance to extremes. Under DT, we observed impairment of aerobic energy metabolism and filtration, as indicated by enhanced pseudofaeces production, even within the thermal niche, which narrows the niche, ultimately lowering LT_50_ by ∼3°C (Götze et al., 2020). Protein damage, blockage of the heat shock response, and constraints on apoptosis may contribute to tissue damage. Our findings thus emphasize the role of the heat shock response and protein replacement in protecting protein and cellular integrity, extending passive thermal tolerance. The range of passive tolerance in bivalve species with different lifestyles (especially contrasting those more and less active) and habitats may be instrumental in securing and predicting survival in marine environments, in light of both progressing and ancient climate changes. Further studies should detail how organismic performance and passive tolerance become constrained, for instance, how the lack of a heat shock response occurs despite the accumulation of damaged proteins.

## Supplementary Material

10.1242/jexbio.250291_sup1 Supplementary information
