# Supplementary material for: Combined climate stressors constrain various mechanisms for thermal tolerance in the scallop Pecten maximus
Source: J Exp Biol. 2025 Dec 15;228(24):jeb250291. doi: 10.1242/jeb.250291 (PMC12752514; doi:10.1242/jeb.250291)
Supplement: Supplementary information [file jexbio-228-250291-s1.pdf]

Table S1. Summary of water chemistry parameters during warming only (W), warming and hypoxia (WHo), warming and hypercapnia (WHc), and deadly trio (DT) exposures. Temperature, salinity, $pH_{Freescale}$, PO_2, and PCO_2, were determined in water samples collected throughout the exposures. Data are presented as means ± SD (n = 5-7 single measurements).

	$T_{nominal}$	T (°C)		Sal (PSU)		$pH_{Freescale}$		PO_2 (mg L^{-1})		PCO_2 (ppm)	
	°C	mean	± sd	mean	± sd	mean	± sd	mean	± sd	mean	± sd
**W**	14	14.35	0.25	34.43	0.26	8.01	0.04	7.94	0.07	571	61
	16	15.61	0.26	33.94	0.46	8.02	0.03	7.54	0.14	619	62
	18	18.22	0.19	34.01	0.53	7.97	0.04	6.97	0.12	644	66
	20	20	0.42	34	0	8.02	0.08	6.45	0.24	695	49
	22	21.97	0.12	33.96	0.25	8.04	0.05	6.68	0.27	591	46
	24	23.97	0.35	34.28	0.58	8.06	0.05	6.29	0.09	597	68
	26	26.3	0.67	33.27	0.66	7.98	0.04	6.31	0.2	701	9
	28	28.37	0.4	34.15	0.07	7.96	0.06	5.95	0.27	769	47
**WHo**	14	13.95	0.09	33.47	0.44	8.10	0.01	4.87	0.23	482	14
	16	16.19	0.34	33.64	0.17	8.07	0.03	4.50	0.20	504	37
	18	18.10	0.23	33.75	0.16	8.12	0.01	4.73	0.14	448	33
	20	20.10	0.28	34.01	0.21	8.04	0.05	4.43	0.08	552	6
	22	21.94	0.32	34.03	0.22	8.05	0.01	4.21	0.19	574	17
	24	24.25	0.23	33.97	0.30	7.97	0.06	4.16	0.24	548	8
	26	26.18	0.05	34.37	0.40	8.01	0.03	4.28	0.17	521	13
	28										
**WHc**	14	13.90	0.07	33.54	0.40	7.51	0.02	8.06	0.02	1750	37
	16	16.27	0.05	33.48	0.10	7.49	0.02	7.67	0.07	1854	18
	18	18.23	0.05	33.72	0.07	7.53	0.01	7.33	0.09	1838	17
	20	20.49	0.06	33.54	0.13	7.53	0.02	6.77	0.14	1798	26
	22	22.30	0.00	33.74	0.13	7.55	0.01	6.65	0.12	1840	49
	24	24.26	0.05	33.94	0.13	7.59	0.00	6.54	0.11	1865	44
	26	26.10	0.20	34.50	0.45	7.55	0.02	6.06	0.07	1818	10
	28	28.72	0.04	34.66	0.54			6.24	0.06	1871	11
**DT**	14	14.34	0.17	33.78	0.9	7.62	0.02	4.58	0.17	1616	78
	16	16.54	0.09	34.05	0.64	7.61	0.01	4.85	0.54	1784	62
	18	17.79	0.06	34.17	0.35	7.67	0.02	4.34	0.2	1882	31
	20	19.84	0.05	33.89	0.25	7.61	0.01	4.3	0.28	1799	29
	22	22.15	0.11	34.11	0.23	7.58	0.01	3.69	0.21	1849	93
	24	23.95	0.62	34.17	0.27	7.54	0.03	3.47	0.23	2025	101
	26	26.31	0.16	33.8	0	7.54	0.03	3.38	0.27	1864	36

Journal of Experimental Biology • Supplementary information

Table S2. Temperature-dependent differences in (A) mean filtration rate, (B) ascending Arrhenius curves of log-transformed resting metabolic rate (RMR), and mantle tissue intracellular pH (pHi). All are summary tables for linear models, with the coefficient for 'treatment' representing the mean difference of warming (W) from DT (the latter being here the reference). (A) shows differences from the reference values at 14°C and of the warming treatment relative to DT. Model uses temperature as a categorical variable and accounts for repeated measures. (B) shows the ascending Arrhenius curve only of log(RMR), covering the shared temperatures of 14 to 20°C and accounting for repeated measures. Treatment does not significantly affect the slope or change the mean log(RMR). Finally, the linear effect of temperature as a continuous variable on pHi is tested for (C) warming-only observations (R^2_{adj} = 0.19) and (D) both warming-only and DT observations (R^2_{adj} = 0.25).

	Coefficients		R	s.e.	DF	t	P
(A) Filtration rate	(Intercept)14		0.74	0.34	31	2.17	0.038
		16	0.47	0.23	31	2.05	0.049
		18	0.32	0.33	31	0.98	0.336
		20	0.44	0.25	31	1.8	0.082
		22	1.06	0.24	31	4.48	<0.001
		24	0.36	0.25	31	1.45	0.158
		26	-0.2	0.33	31	-0.59	0.561
		28	-0.5	0.39	31	-1.27	0.213
	TreatmentW		-0.37	0.42	7	-0.89	0.403
(B) log(RMR) ascending Arrhenius curves	(Intercept)		39.08	6.69	33	5.84	<0.001
	Temperature (1000/K)		-10.54	1.94	33	-5.44	<0.001
	TreatmentW		-7.83	8.72	12	-0.9	0.387
	Temperature:TreatmentW		2.27	2.53	33	0.9	0.377
(C) Warming-only coefficients	(Intercept)			7.022	0.055	127.81	<2e-16
	Temperature (°C)			-0.007	0.003	-2.49	0.0212
(D) Warming-only and DT coefficients	(Intercept)			7.069	0.048	146.681	< 2e-16
	Temperature (°C)			-0.009	0.002	-4.008	0.0002

Journal of Experimental Biology • Supplementary information

Table S3. ANOVA tables for linear models assessing the temperature-dependence of *P.maximus* responses. Also shown are differences between warming-only and DT treatments, and any interaction between temperature (°C) and treatment. Responses include (A) haemolymph P_eO_2 (Fig. 3), (B) intracellular pH (pHi; Fig. 4), (C) gill tissue malondialdehyde (MDA; Fig. 5), (D) gill tissue heat shock protein 70 content (HSP70; Fig. 6), (E) gill tissue ubiquitin, (F) gill tissue Branchial Branched Chain Amino Acid concentrations (BCAA; both in Fig. 7), and (G) gill tissue activity of caspase-3 and -7 (Fig. 9). Analysis in (E) and (F) is of log-transformed data

	Source of Variation	DF	SS	MS	F	P
(A) Haemolymph P_eO_2	Temperature	3	71.74	23.91	11.13	<0.001
	Treatment	1	108.05	108.05	50.31	<0.001
	Temperature x Treatment	3	0.07	0.02	0.01	0.998
	Residual	35	75.17	2.15		
	Total	42	244.71	5.83		
(B) pHi	Temperature	3	0.15	0.05	13.27	<0.001
	treatment	1	0	0	0.87	0.356
	Temperature x Treatment	3	0.01	0	0.64	0.593
	Residual	37	0.14	0		
	Total	44	0.3	0.01		
(C) Gill MDA	Temperature	3	440075.7	146691.9	15.05	<0.001
	Treatment	3	252115.6	84038.53	8.62	<0.001
	Temperature x Treatment	9	67998.85	7555.43	0.78	0.64
	Residual	74	721329.2	9747.69		
	Total	89	1422564	15983.87		
(D) Gill HSP70	Temperature	3	6.23	2.08	14.93	<0.001
	Treatment	3	4.49	1.5	10.75	<0.001
	Temperature x Treatment	9	3.27	0.36	2.61	0.012
	Residual	70	9.74	0.14		
	Total	85	24.13	0.28		
(E) Gill ubiquitin	Temperature	3	1.41	0.47	9.75	<0.001
	Treatment	3	5.05	1.68	34.8	<0.001
	Temperature x Treatment	9	3.3	0.37	7.58	<0.001
	Residual	74	3.58	0.05		
	Total	89	13.07	0.15		
(F) Gill BCAA	Temperature	3	1.91	0.64	3.04	0.034
	Treatment	3	0.52	0.17	0.83	0.482
	Temperature x Treatment	9	3.37	0.37	1.79	0.085
	Residual	76	15.92	0.21		
	Total	91	21.5	0.24		
(G) ORAC	Temperature	30	0.36	0.01	1.77	0.173
	Treatment	1	0.28	0.28	42.44	<0.001
	Temperature x Treatment	30	0.47	0.02	2.32	0.095
	Residual	30	0.20	0.01		
	Total	37	0.58	0.02		
(H) Gill caspase	Temperature	3	5.18	1.73	16.42	<0.001
	Treatment	3	3.83	1.28	12.15	<0.001
	Temperature x Treatment	9	3.49	0.39	3.69	<0.001
	Residual	75	7.88	0.11		
	Total	90	20.56	0.23		

Journal of Experimental Biology • Supplementary information
